# Deep Whole-Genome Sequencing to Detect Mixed Infection of *Mycobacterium tuberculosis*

**DOI:** 10.1371/journal.pone.0159029

**Published:** 2016-07-08

**Authors:** Mingyu Gan, Qingyun Liu, Chongguang Yang, Qian Gao, Tao Luo

**Affiliations:** 1 Key Laboratory of Medical Molecular Virology of Ministries of Education and Health, Institutes of Biomedical Sciences and Institute of Medical Microbiology, School of Basic Medical Sciences, Fudan University, Shanghai, China; 2 Laboratory of Infection and Immunity, School of Basic Medical Science, West China Center of Medical Sciences, Sichuan University, Chengdu, Sichuan, China; St. Petersburg Pasteur Institute, RUSSIAN FEDERATION

## Abstract

Mixed infection by multiple *Mycobacterium tuberculosis* (MTB) strains is associated with poor treatment outcome of tuberculosis (TB). Traditional genotyping methods have been used to detect mixed infections of MTB, however, their sensitivity and resolution are limited. Deep whole-genome sequencing (WGS) has been proved highly sensitive and discriminative for studying population heterogeneity of MTB. Here, we developed a phylogenetic-based method to detect MTB mixed infections using WGS data. We collected published WGS data of 782 global MTB strains from public database. We called homogeneous and heterogeneous single nucleotide variations (SNVs) of individual strains by mapping short reads to the ancestral MTB reference genome. We constructed a phylogenomic database based on 68,639 homogeneous SNVs of 652 MTB strains. Mixed infections were determined if multiple evolutionary paths were identified by mapping the SNVs of individual samples to the phylogenomic database. By simulation, our method could specifically detect mixed infections when the sequencing depth of minor strains was as low as 1× coverage, and when the genomic distance of two mixed strains was as small as 16 SNVs. By applying our methods to all 782 samples, we detected 47 mixed infections and 45 of them were caused by locally endemic strains. The results indicate that our method is highly sensitive and discriminative for identifying mixed infections from deep WGS data of MTB isolates.

## Introduction

Tuberculosis (TB) remains a great threat to human healthy by causing around 9.6 million new cases and 1.5 million deaths in 2014 [1]. TB is usually caused by infection of a single strain of *M*. *tuberculosis* (MTB), but molecular genotyping methods have proven that a patient could be infected with multiple genetically distinct strains, which we refer to as “mixed infection” [2–4]. Mixed infections could lead both clinical and public problems. Mixed infections with both drug sensitive and resistant strains can lead to discordant drug-susceptibility test profiles, which could complicate the treatment regimen and lead to poor treatment outcomes [2, 5, 6]. Mixed infections could also lead to underestimation of the ongoing transmission of MTB. It is possible that only one strain from a patient of mixed infection is transmitted to a secondary patient. If this strain were not identified from the index case, the transmission would be ignored [7]. The frequency of mixed infection may differ according to the level of MTB transmission in different areas. If mixed infection is common in a given population, a high rate of recent transmission may be indicated.

Mixed infection can be detected by traditional genotyping methods, such as spoligotyping, *IS6110* restriction fragment length polymorphism (RFLP), and variable-number tandem repeat (VNTR). Based on such methods, the rates of mixed infection were found ranged from 10–20% in high TB incidence areas [4, 8, 9]. However, the estimated rate based on mathematical model is much higher than we detected [10], which suggest the sensitivity of these methods are limited. Spoligotyping based detection has the limitation of low resolution as it targets a single locus of the MTB genome [11]. Furthermore, it is difficult do differentiate the spoligotype pattern of mixed strains from that of a single strain when the spoligotype patterns of local strains are similar [8]. The detection of mixed strains by *IS6110* RFLP is mainly based on the identification of hybridizing low-intensity bands, which prone to be subjective [12]. VNTR-based detection of mixed infection depends on the identification of multiple bands in one or more VNTR loci. The problem of VNTR typing is that it is difficult to distinguish mixed infections from clonal heterogeneity [13]. Finally, all above methods have limited sensitivities to detected low-abundance DNA from the minor strain in a mixed infection. An abundance of 10% of the minor strain is usually needed to achieve an unambiguous detection by these methods [12, 14]. Recently, some PCR-based methods that target lineage specific markers (i.e., *IS6110* insertion, large fragment deletions) could achieve much higher detection limit of the minor strain. However, the resolution of such methods is low, as they could only detect mixed infections by certain MTB lineages/sublineages [4].

Deep whole genome sequencing, which is based on the next generation sequencing technology, provides ultimate resolution for typing MTB [8]. Single nucleotide variations (SNVs) detected by mapping sequencing reads to a reference genome were usually used to illustrate the genomic distance between MTB isolates [15–17]. Clinical MTB isolates from solid/liquid cultures are mixture of many bacterial colonies multiplied from the original sputum, thus the deep sequencing data of such samples contains information refer to the genetic diversity of the within host bacterial population. Since MTB is haploid, the existence of an extraordinarily large number of high-quality heterogeneous SNVs may suggest a potential mixed infection [18]. E.g., Köser CU. et al., identified 209 heterogeneous SNVs from an early positive liquid (MGIT) culture and they proved that the patient was infected by two distantly related Beijing strains [19]. Chan J. et al., applied metagenomic analysis to a sample from a 215-year old mummy and identified 398 heterogeneous SNVs after mapping the sequencing data to the genome of H37Rv, which indicates the person had a mixed infection of MTB [20]. However, due to the interference of sequencing error, the calling of high-quality heterogeneous SNVs usually needs relatively high abundance of the minor strain (i.e., >30%) in a mixed infection [18, 19]. Furthermore, only mixed infections caused by genetically distantly related strains (i.e., genomic difference >100 SNVs) could be unambiguously detected by such methods. When only a small number of heterogeneous SNVs are detected, it will be less accurate to tell the heterogeneity is caused by mixed strains or by microevolution after infection [18]. In this study, by taking the advantage of next generation sequencing, we developed a phylogenetic-based method that could achieve a sensitive and discriminative detection of mixed infection of MTB.

## Materials and Methods

### WGS data collection and SNV detection

We downloaded the WGS data of 782 strains from NCBI SRA database (S1 Table) [16, 21–26]. The reads of each strain were extracted by Fastq-dump of SRA Toolkit (v2.5.7). The low quality reads were trimmed with Sickle (https://github.com/ucdavis-bioinformatics/sickle). The trimmed reads were then mapped against the artificial genome of the most recent common ancestor (MRCA) of MTB (MTB_*mrca*_) [27] with Bowtie2 (v2.1.0). SNVs were identified using SAMtools/BCFtools (v0.1.18) and VarScan (v.2.3.6).

We used two approaches, SAMtools/BCFtools (v0.1.18) and VarScan (v.2.3.6) for SNV calling. SAMtools/BCFtools was used to call homogeneous SNVs (variant allele frequency ≥95%), while VarScan was used to call both homogeneous and heterogeneous SNVs (variant allele frequency <95%) from the pileup files generated by SAMtools. In both cases, SNVs were called at loci with minimum depth of 15, minimum mapping quality of 20 and minimum base quality of 20. By SAMtools/BCFtools, SNVs were further filtered according to the variant frequency (≥ 95%). By VarScan, SNVs were called if they were supported by at least two reads and passed the strand-bias filter at the same time. SNVs in PE/PPE/PGRS genes and transposons were excluded to avoid any concern about inaccuracies in the read alignment in those portions of the genome. Furthermore, SNVs in an additional 39 drug-resistance associated genes [28] were also removed to exclude the possibility that homoplasy of drug resistance mutations would significantly decrease the reliability of phylogeny.

### Strain filtering and phylogenomic database construction

Homogeneous SNVs called by both SAMtools/BCFtools and VarScan were used for constructing a phylogenomic database. Firstly, we excluded strains with significantly lower or higher number of homogeneous SNVs using the methods described by Tukey JW [29]. Since MTB strains of the same lineage accumulated similar number of SNVs from the MTB_*mrca*_ [24], an extraordinary small or large number of SNVs may indicate artifacts in mapping or variant calling, which may influence the reliability of phylogeny. In total, 58 outlier strains were excluded (S1 Fig). The remaining 724 strains were further defined as clustered/unique strains by pair-wise comparison. We found 124 strains were grouped into 52 genomic clusters (difference ≤10 SNVs within cluster). We randomly selected one genome from the clustered strains, resulted in 652 strains represented as unique genomes. We included all the unique genomes for phylogenomic database construction.

Homogeneous SNVs of the selected 652 strains were combined into a non-redundant SNV list. According to this list, we recovered the base calls for each strain and combined them into a concatenated alignment. We filtered SNV loci that with a frequency of missing data (caused by indels, low coverage or low mapping quality) >5%. The filtered alignment was then used to generate a maximum likelihood (ML) phylogeny by RaxML (v8.0.20) using the GTR nucleotide substitution model. A joint ancestral sequence reconstruction of each node was inferred with HyPhy (2.22). Branch specific SNVs were identified by comparing descendent nodes with the closest ancestral node.

### Description of the phylogenetic-based algorithm

The algorithm was designed to detect evolutionary path(s) according to the homogeneous and heterogeneous SNVs of a sample. Mixed infection was determined as if two or more evolutionary paths were detected. The algorithm includes four main steps as follows (S2 Fig).

1. Map the homogeneous and heterogeneous SNVs (called by VarScan) of a sample to the phylogenomic database according to the position and allelic change of each SNV locus and record the mapped branches (S2A Fig).
2. Exclude mapped branches whose coverage (defined as the proportion of the matched SNVs in a branch) are lower than 10% and keep the others as candidate branches (S2B Fig).
3. Extract the evolutionary routes for all candidate branches from the database and assemble them into candidate paths by pairwise comparison, during which the shorter routes were merged with the longer ones that can fully cover them (S2C Fig).
4. For every combination of two candidate paths, determine the diversification node, the shared segment and unique segments of them (S2D Fig). Then, identify authentic mixed infections by following criteria:
  - For the branches in each segment (the shared segment and two unique segments), at least 60% of them are mapped with coverage ≥10%.
  - The three branches connected by the diversification node should be all mapped.

### Generate synthetic reads for single or mixed MTB strains

We generated artificial genomes for 500 of the 652 strains in the phylogenomic database according to their homogeneous SNVs. For each strain, we created its synthetic genome based on the genome of MTB_*mrca*_ by replacing the ancestral bases with corresponding mutant bases in the SNV loci. The synthetic genome was then used to generate artificial illumina paired-end reads (with a depth of 100) using ART (v03.09.15), which simulates base quality and sequencing errors by emulating the sequencing process with built-in base quality value profiles and read error models. For the simulation of mixed sample, we selected 25 pairs (S2 Table) of strains from the 500 strains. The genomic distance between strains in each pair ranged from 10 to 1621 SNVs. To monitor mixed infections at different levels, we mixed the simulated reads of the paired strains in different ratios, in which the depth of the minor strain was set as 1×, 2×, 3×, 5× or 10×, with corresponding depth of the major strain as 99×, 98×, 97×, 95×, or 90× respectively to guarantee a total depth of 100×. Similarly, we further generated mixed reads for 52 pairs of MTB strains that were not included in the phylogenomic database (S3 Table). The synthetic reads of all non-mixed and mixed samples were then mapped to the genome of MTB_*mrca*_ and SNVs were called by VarScan as mentioned above.

### Detection of mixed infection

The genomic SNVs (including both homogeneous and heterogeneous ones) called by VarScan from clinical or artificial samples were used as inputs for detecting mixed infections. The non-mixed samples of 500 individual strains were used to test the specificity of our methods. The mixed samples of paired strains were used to test the detecting sensitivity (defines as the limit of depth of the minor strain) and resolution (defines as the limit of genomic distance between two mixed strains) of our method.

## Results

### Principle of the phylogenetic-based detection of mixed infection

**Fig 1:**
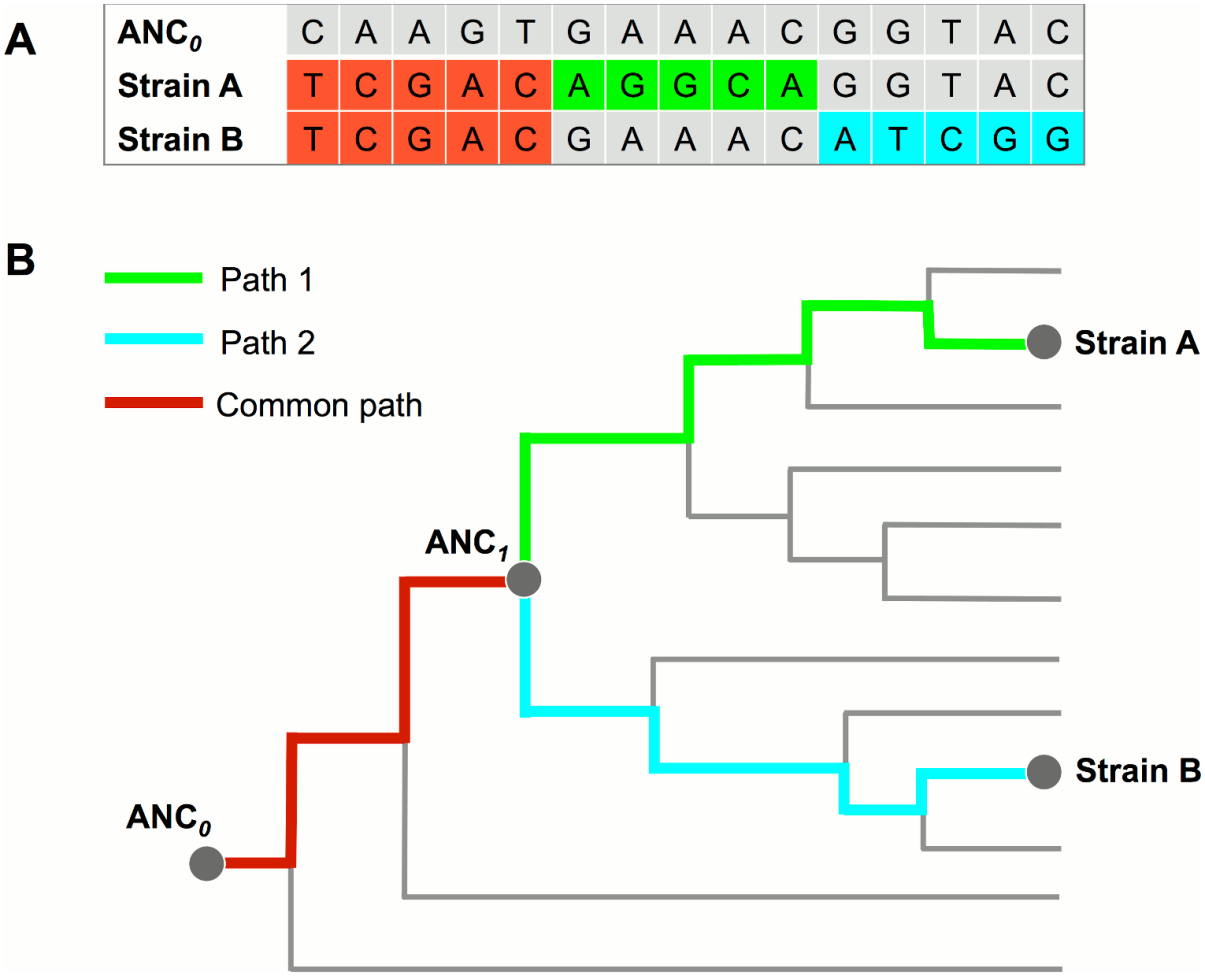
Principle of the phylogenomic-database based detection of mixed infection. **(A)** WGS reads of a mixed infection by strain A and B were mapped to the ANC_*0*_ genome to call SNVs that include common SNVs (red color) shared by both strains and strain specific SNVs (green and blue color). **(B)** The evolutionary paths of the two strains were determined by mapping SNVs to the reference phylogenomic database. These two paths share a common segment (mapped by common SNVs) from ANC_*0*_ to ANC_*1*_ and diverged into two separated segments (mapped by strain specific SNVs) after ANC_*1*_.

MTB is a clonal bacterial pathogen whose evolution is mostly a process of stepwise accumulation of genomic mutations [30]. The evolutionary path of a MTB strain could be determined by mapping its genomic mutations onto a reference phylogeny, computed from pre-existing MTB genomes. We defined the MRCA of MTB as ANC_*0*_ in the reference phylogeny (Fig 1), and defined the MRCA of two MTB strains in a mixed infection as ANC_*1*_. We called SNVs of each sample by mapping the sequencing reads to the genome of ANC_*0*_. The phylogenetic-based algorithm was designed to map SNVs to the reference phylogeny and detect potential evolutionary path(s). If two paths are identified, mixed infection will be determined (Fig 1). These two paths shared a common evolutionary segment from ANC_*0*_ to ANC_*1*_ and diverged into two segments from ANC_*1*_.

### The reference phylogenomic database of global MTB

**Fig 2:**
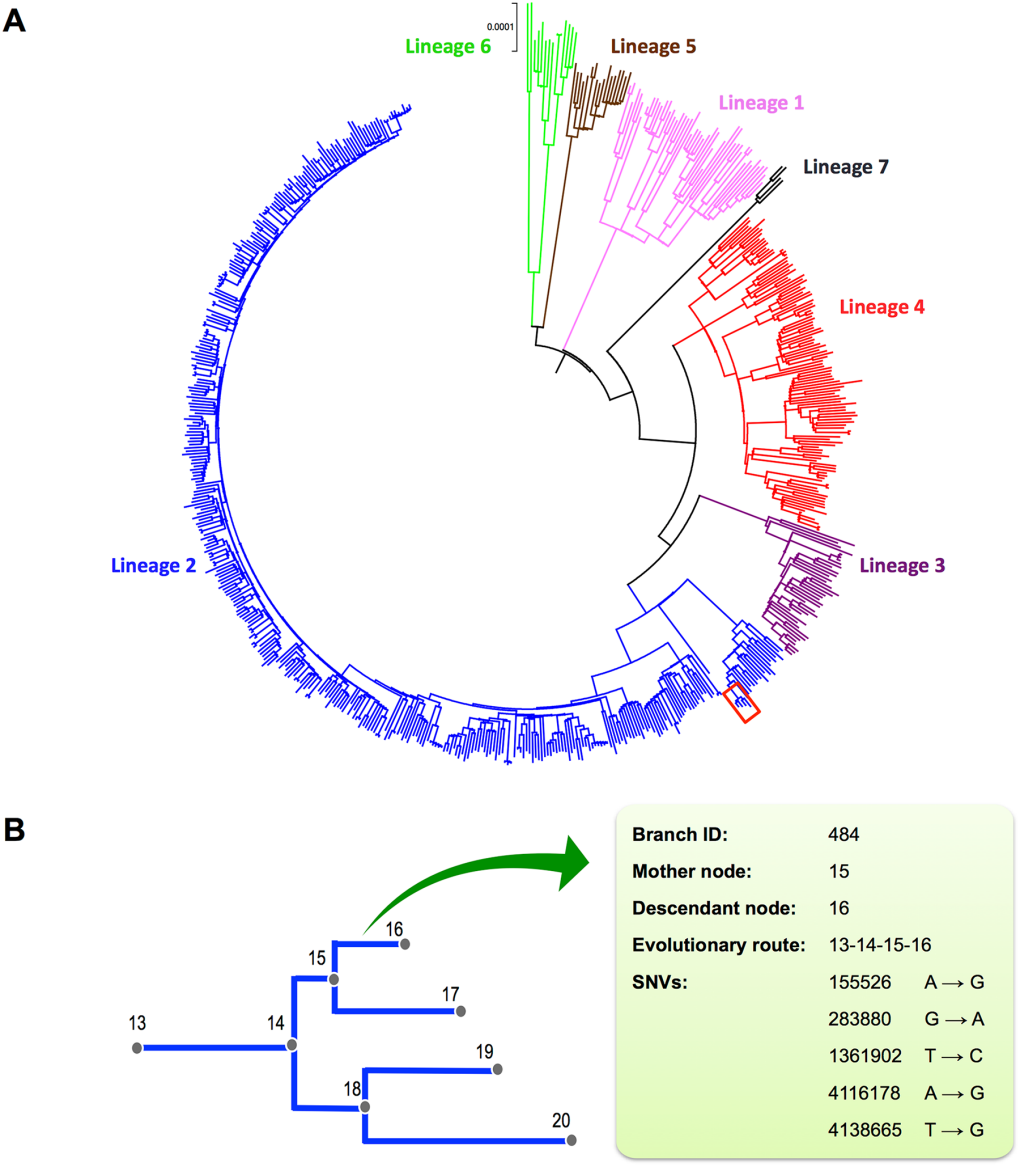
The phylogenomic database of global MTB. **(A)** Maximum-likelihood (ML) phylogeny of 652 global MTB strains based on the concatenated alignment of 68,639 genomic SNVs. The colors represent seven MTBC lineages. **(B)** Schematic diagram illustrating the components of the phylogenomic database. The nodes of the ML phylogeny (represented by a sub-branch outlined in red in panel A) were numbered to record the branching order. A branch was determined by its mother and descendant nodes. The SNVs and evolutionary route of the each branch were recorded.

By excluding SNVs in drug resistance genes, mobile elements and PE/PPE family genes, a total of 68,639 homogeneous SNVs of the 652 MTB strains collected worldwide were used to construct a reference phylogenomic database (S1 Text). Firstly, we constructed a maximum likelihood phylogeny based on the concatenated alignment of these strains (Fig 2A). According to the phylogeny, global MTB consists of seven major lineages, which is consistent with previous studies [31]. Secondly, we inferred the maximum likelihood sequence of each ancestral node. Thirdly, we inferred the branch specific SNVs by comparing the reconstructed sequence of the descendant node to the closest ancestral node. Excluding the *M*. *canettii* (the out-group), the phylogeny consists of 1,313 branches with a median branch length of 41 SNVs (range from 1 to 840 SNVs). The branching events along the phylogeny, together with corresponding nucleotide changes, compose the reference phylogenomic database (Fig 2B).

### Simulation of artificial sequencing data

**Fig 3:**
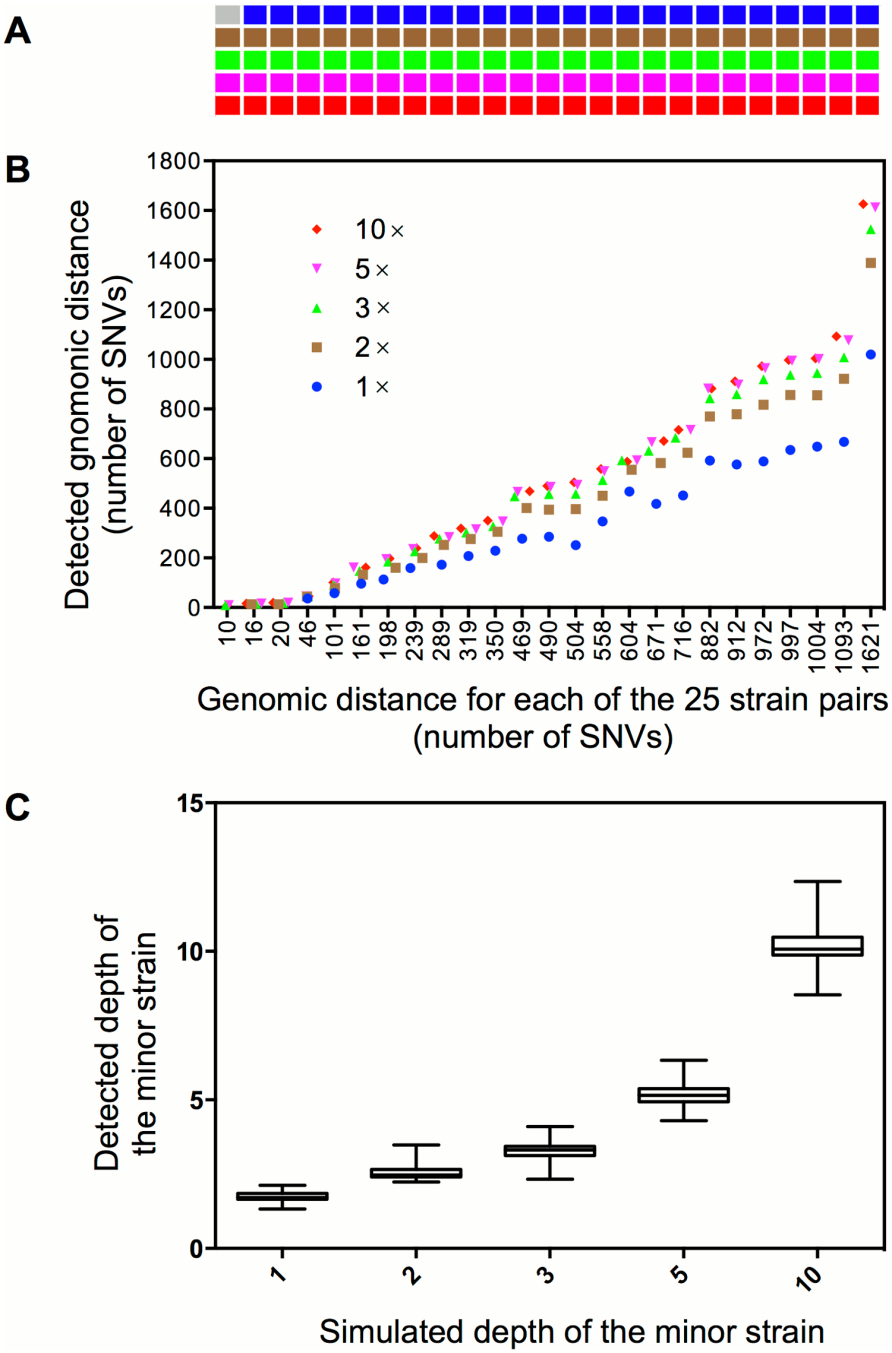
Detection of mixed infections from artificial WGS reads. **(A)** The cells represent 125 synthetic samples of mixed infection. Samples in the same column represent five different levels of mixed infections (depth from top to bottom is 1×, 2×, 3×, 5× or 10× for the minor strain) that are synthesized from the same pair of strains whose genomic distances are in correspondence with the X-axis of panel B. The gray cell indicates no detection of mixed infection and the cells in other five colors indicate detection of mixed infections in different mixed levels. **(B)** The detected genomic distance (defines as number of SNVs) between the mixed strains in each synthetic sample. **(C)** The estimated depth of the minor strain under the five simulated depths.

Synthetic mixed infections with 25 strain pairs that differ in abundance and/or genomic distance were generated to test the sensitivity and resolution of our method (Fig 3A). Among the 125 simulations, 124 (99.2%) were successfully detected as mixed infections (Fig 3A). The detected genomic distance between two mixed strains is close to their real distance when the depth of the minor strain is higher than 1× (Fig 3B); most of the detected depth of two strains in each simulation is congruent with the original simulated depth (Fig 3C). For the only one sample that failed to be detected as mixed infection, it was mixed by a strain pair that has the smallest genomic distance (10 SNVs) and the lowest depth (1×) of the minor strain.

To test the specificity of our methods, we applied the analysis to the non-mixed reads of 500 individual strains. No mixed infection was detected from these samples, which demonstrated a specificity of 100%.

### Detecting mixed infection from clinical samples

Among all 782 WGS data, 15 were from single-clone MTB culture [32]. By applying our methods to these data, no mixed infection was detected. For the remaining 767 samples of multi-clone MTB culture, we detected potential mixed infection from 47 samples (47/767, 6.1%) (Table 1). The depth of the minor strains in these samples ranged from 2.13× to 100.40×, and the proportion of the minor strains ranged from 0.64% to 35.83%. Of the detected mixed infections, 21 cases were caused by two strains of different MTB lineages. For these cases, an average of 825.8 (from 361 to 1,735) SNVs were mapped to the two strain specific paths. For the remaining 26 cases, infections were caused by two strains of the same lineage (Lineage 2 or 4) and an average of 222 (61–655) SNVs were mapped to the two strain specific paths (Table 1). In most cases (45/47, 95.74%), the two strains of a mixed infection were belonging to local endemic genotypes (Table 1). E.g., mixed infections in Shanghai were all caused by lineage 2 and/or lineage 4 strains.

**Table 1:** Clinical samples detected as mixed infection by our methods.

Sample ID	Origin	Minor strain		Major strain		#Coverage
		Depth (abundance)	Lineage	Depth (abundance)	Lineage	
SRR671847	Guizhou, China	2.13(1.92%)	2	90.45(98.78%)	2	655/833(78.63%)
SRR671801	Liaoning, China	2.20(1.70%)	4	98.30(99.33%)	2	700/794(88.16%)
855Bb4	Nepal	2.21(1.65%)	4	131.82(99.18%)	3	739/1016(72.74%)
10–390	Shanghai, China	2.25(0.93%)	2	241.13(99.39%)	2	87/111(78.38%)
N0130b4	San Francisco	2.33(1.33%)	2	156.71(99.43%)	2	100/129(77.52%)
SRR671835	Guizhou, China	2.35(2.10%)	2	89.04(98.94%)	4	361/916(39.41%)
SRR671811	Shanghai, China	2.37(2.10%)	2	97.21(98.79%)	2	301/501(60.08%)
*N0182	London/Homerton	2.39(1.91%)	2	99.01(98.35%)	1	1145/1555(73.63%)
12–1813	Shanghai, China	2.45(1.09%)	2	195.79(99.38%)	4	366/949(38.57%)
09–783	Shanghai, China	2.46(0.91%)	2	241.38(99.75%)	4	363/984(36.89%)
SRR671725	Guangdong, China	2.46(2.02%)	2	99.20(98.93%)	4	376/786(47.84%)
SRR671824	Shanghai, China	2.50(2.42%)	2	84.95(99.17%)	2	268/501(53.49%)
DY21	Ghana	2.61(5.11%)	1	46.65(97.04%)	5	1182/1703(69.41%)
12–1828	Shanghai, China	2.61(1.13%)	4	203.33(99.42%)	2	697/944(73.83%)
GX94	Guangxi, China	2.64(2.07%)	2	103.49(99.27%)	2	134/255(52.55%)
09–0645	Shanghai, China	2.67(1.11%)	2	186.37(99.28%)	2	105/131(80.15%)
MTB_DY_131	Ghana	2.67(2.95%)	1	79.87(97.97%)	4	624/1612(38.71%)
09–0645_cut	Shanghai, China	2.70(1.02%)	2	210.61(99.11%)	2	107/131(81.68%)
GX187	Guangxi, China	2.73(2.88%)	2	77.69(97.49%)	2	246/423(58.16%)
09–1780	Shanghai, China	2.75(0.64%)	2	414.00(99.77%)	2	221/287(77.00%)
GQ-1165	Shanghai, China	2.75(0.89%)	2	274.19(99.23%)	2	100/167(59.88%)
*N0041b4	San Francisco	2.75(1.99%)	6	137.20(98.86%)	2	994/1764(56.35%)
12_0358	Shanghai, China	3.13(0.83%)	4	376.60(99.51%)	2	693/867(79.93%)
11–1912	Shanghai, China	3.16(0.85%)	2	313.21(99.67%)	2	169/305(55.41%)
11–2094	Shanghai, China	3.33(1.12%)	2	301.31(99.49%)	2	143/204(70.10%)
11_1549	Shanghai, China	3.33(1.37%)	2	207.83(99.63%)	2	105/112(93.75%)
10–360	Shanghai, China	3.43(0.92%)	2	332.15(99.45%)	2	122/193(63.21%)
SRR671837	Guizhou, China	3.58(3.21%)	2	93.37(96.06%)	2	61/69(88.41%)
09–1687	Shanghai, China	3.75(0.79%)	2	390.36(99.36%)	2	241/302(79.80%)
N0185	London	3.93(3.82%)	2	81.57(93.97%)	4	589/835(70.54%)
10–1563	Shanghai, China	4.40(2.38%)	2	165.95(98.91%)	2	144/241(59.75%)
09–1060	Shanghai, China	4.42(2.40%)	2	175.61(97.23%)	4	687/826(83.17%)
SRR671840	Tibet, China	4.64(3.97%)	2	90.87(99.04%)	2	414/784(52.81%)
SRR671866	Beijing, China	5.34(4.88%)	2	88.64(94.04%)	2	120/130(92.31%)
10–592	Shanghai, China	6.16(1.56%)	4	345.88(97.95%)	2	947/1012(93.58%)
BTBS517	Ethiopia	6.50(6.86%)	4	65.61(87.61%)	6	1326/1535(86.38%)
09–799	Shanghai, China	6.65(1.58%)	2	354.19(97.08%)	2	304/334(91.02%)
09–716	Shanghai, China	6.83(1.64%)	2	373.92(98.01%)	2	270/283(95.41%)
751Bb4	Nepal	7.88(5.86%)	2	119.98(93.07%)	3	846/865(97.80%)
MTB_5_V367IO	Vietnam	17.62(5.39%)	1	271.46(93.34%)	4	1300/1434(90.66%)
N0089b4	The Gambia	18.37(14.45%)	2	103.06(84.24%)	6	1735/1764(98.36%)
10_0841	Shanghai, China	24.24(10.97%)	2	167.65(84.27%)	4	826/870(94.94%)
D2g0841	Shanghai, China	36.28(9.42%)	2	296.56(85.47%)	4	845/959(88.11%)
09–682	Shanghai, China	37.86(15.28%)	2	226.76(99.75%)	2	129/306(42.16%)
N0138b4	San Francisco	51.71(30.73%)	4	112.67(67.61%)	4	526/543(96.87%)
11–220	Shanghai, China	90.44(35.83%)	2	137.08(56.34%)	2	350/353(99.15%)
10–2065	Shanghai, China	100.40(32.88%)	2	177.05(59.57%)	2	351/353(99.43%)

*One of the mixed strains is not belonging to the local endemic genotype.

^#^Defined as the ratio between the detected number of SNVs and the real number of SNVs of two mixed strains.

## Discussion

MTB mixed infection would complicate the treatment regime and interfere the resistance profile detecting [2]. In this study, we developed a phylogenetic-based method for detecting mixed infection based on WGS data of MTB culture. Our method is based on a reference phylogenomic database, which could not only overcome bias caused by false positive SNV callings, but also differentiate heterogeneity caused by mixed infections or within-host microevolutions. Such features make our method highly discriminative that could detect mixed infection by two strains with very small genetic distance. The method is also highly sensitive that could detect minority strains with sequencing depth as low as 1×.

Several recent researches reported mixed infections of MTB based on the identification of hundreds of heterogeneous SNVs identified from NGS data [19, 20]. The heterogeneous SNVs called from deep sequencing data in this case could be resulted from PCR/sequencing errors or newly evolved mutations through microevolution after infection, which could confounds the identification of rare alleles of the minor strain in a mixed infection. Consequently, when the frequency of the minor strain is high (i.e., >30%), mixed alleles would be easily identified. Contrarily, if the minor strain is less frequent, it will be unpractical to distinguish mixed alleles from other rare variants. Furthermore, as a number of newly evolved mutations could be selected or drifted to a high frequency within patient [25], mixed infection could be determined only when the mixed strains are genetically distantly related (e.g., genomic difference >100 SNVs). Our methods successfully exclude the interference of PCR/sequencing errors and newly evolved mutations through mapping SNVs to the phylogenomic database. Since the database is constructed based on homogeneous SNVs that have been fixed in clinical strains, mutations newly evolved after infection will not likely to be mapped to the reference phylogeny. As for PCR/sequencing errors, there are very rare chances they would be mapped to the phylogeny. For the ones that are mapped to the phylogeny, they should appear as sporadic on the tree and there is a small likelihood to observe a series of adjacent branches mapped by such SNVs. Thus, the corresponding paths will be filtered by our algorithm.

Recently, several methods have been developed for detecting mixed infection based on deep sequencing data. David et al. developed a maximum likelihood-based method by estimating the proportion of major strain and divergent sites [33]. Sergio et al. developed a method by constructing the haplotypes of mixed strains [34]. However, by applying these two methods to the data of 15 MTB single-colony samples, we found both methods incorrectly identified mixed infections in all of them. Since the influence of sequencing error has been largely excluded in both methods, the false-positive results may be caused by the microevolution, through which newly evolved mutations may be selected or drifted to a frequency that could be identified by deep sequencing.

By our method, the detection of mixed infection requires just 1× read depth from the minority strain. According to the simulation results, when the genetic distance between two strains is higher than 16, mixed infections can be constantly detected when the depth of the minor strains is only 1×. For the 47 clinical specimens detected as mixed infections by our method, the lowest proportion of minority strain is 0.64%, which demonstrated our method is much more sensitive than current genotyping-based detections (e.g. VNTR based detection requires a proportion of more than 10% of the minor strain).

Of the mixed infections detected in clinical samples, most (45/47) of the mixed strains belong to the local endemic genotype, which demonstrates a high reliability of our method. For the two exceptions, both cases are associated with immigrant (Table 1). Sample N0182 was isolated from London and it was found as a mixed infection by strains of Lineage 1 and Lineage 2. The corresponding patient was born in Malaysia where Lineage 1 and Lineage 2 strains are endemic [35]. So it is possible that this patient was infected with these two strains before migration. The other exception is sample N0041b4 that was isolated from San Francisco and was found mixed with Lineage 6 and Lineage 2 strains. MTB Lineage 6 was not an endemic genotype in San Francisco or in the born place (Vietnam) of the patient. However, as San Francisco is a city of migrants, it’s possible that this patient had been re-infected with a Lineage 6 strain from the West African migrants.

The detection of mixed infection by our method is depending on the identification of divergence event (i.e., two strain specific paths) of two mixed strains whose strain specific paths are both completely or partially included in the database (S3A–S3C Fig). Therefore, if the divergence of two strains is not included in the database (S3D and S3E Fig), mixed infection will be missed. In the current study, we detected almost all of the mixed infections simulated by 25 pairs of strains selected form the database (Fig 3). In contrast, by applying our method to simulated data of separate MTB genomes (genomes not included in the reference database, S3 Table), we found mixed infections by strains of different lineages could be all detected, while many mixed infections by strains of the same lineage were missed (S4 Fig). The undetected mixed infections mainly belonged to pairs of strains with small genomic differences (i.e., <200 SNVs, S4 Fig), in which cases the divergence of two strains happened more recently and was not included in the database (data not shown). Taken together, a comprehensive reference database is necessary for our phylogenetic-based detection. Since it is impossible to cover all the genetic diversity of global MTB, constructing local databases that contain both remote and recent divergence events of MTB strains in local areas would be an applicable strategy. Moreover, detecting in a recursive way would also increase the sensitivity of our method. As described in current study, we firstly constructed the phylogenomic database using the homogeneous SNVs of the clinical samples, and then we mapped both heterogeneous and homogeneous SNVs of these samples for detection of mixed infections. Similarly, one can integrate homogeneous SNVs of target samples to an existing reference database and then perform the detection. By such strategy, the sensitivity of our method could be guaranteed.

In conclusion, we developed a phylogenetic-based method that could achieve a sensitive and discriminative detection of MTB mixed infection from WGS data. As WGS has been increasingly used for studying the epidemiology of TB, more and more sequencing data of MTB from local areas will be available. Our method provides a solution to inspect mixed infections from those data and enables to gain a further insight into the local TB epidemic.

## Supporting Information

S1 Fig Exclusion of the outlier strains based on analyzing the SNV number of different MTB lineages. (TIF)

S2 Fig Schematic diagrams illustrating the procedure of determining mixed infection by the phylogenetic-based method. **(A)** Map the SNVs (red vertical lines) of a sample to the reference phylogeny. **(B)** Exclude branches with low SNV coverage (<10%). **(C)** Assemble mapped branches into candidate evolutionary paths (black thick lines). Strings above/below branches indicate evolutionary routes of corresponding branches. **(D)** Determine authentic pairs of paths (colored lines) from all possible combinations.
(TIFF)

S3 Fig Schematic diagrams illustrating possible cases of mapping the evolutionary paths of two MTB strains to the reference phylogenomic database. The two unique paths after divergence are completely **(A)**, or partially **(B, C)** covered by the database. In other cases, one **(D)** or both **(E)** unique paths are not covered by the database. Thick colored lines represent path segments covered by the database. Red dash lines represent path segments not included in the database.
(TIFF)

S4 Fig Detection of mixed infections from mixed reads simulated with MTB genomes not included in the phylogenomic database. Three panels represent simulated mixed infections by strains within Lineage 2 **(A)** or Lineage 4 **(B)**, or by strains of different lineages **(C)**. For each panel, the numbers above the first row represent the genomic distance between two strains. The numbers left to the first column represent the depth of the minor strains. The gray and blue cells indicate failure and success of detection respectively.
(TIFF)

S1 Table List of 782 strains downloaded from NCBI SRA database with information of MTBC lineage, isolation place and mapping information. (XLSX)

S2 Table List of the 25 pairs of strains used for simulating mixed infections. (XLSX)

S3 Table List of strains separated from our database. These 52 strain pairs were used for simulating mixed infections.
(XLSX)

S1 Text The phylogenomic database constructed based on 652 global MTB strains. The database composes 1,313 branches that have been numbered from the root. For each branch, the closest ancestral branch, the evolutionary route from the root, the branch level, the genomic coordinates and nucleotide of corresponding SNVs were recorded.
(TXT)
