# Supplementary figures and images for: Deep Whole-Genome Sequencing to Detect Mixed Infection of Mycobacterium tuberculosis
Source: PLoS One. 2016 Jul 8;11(7):e0159029. doi: 10.1371/journal.pone.0159029 (PMC4938208; doi:10.1371/journal.pone.0159029)

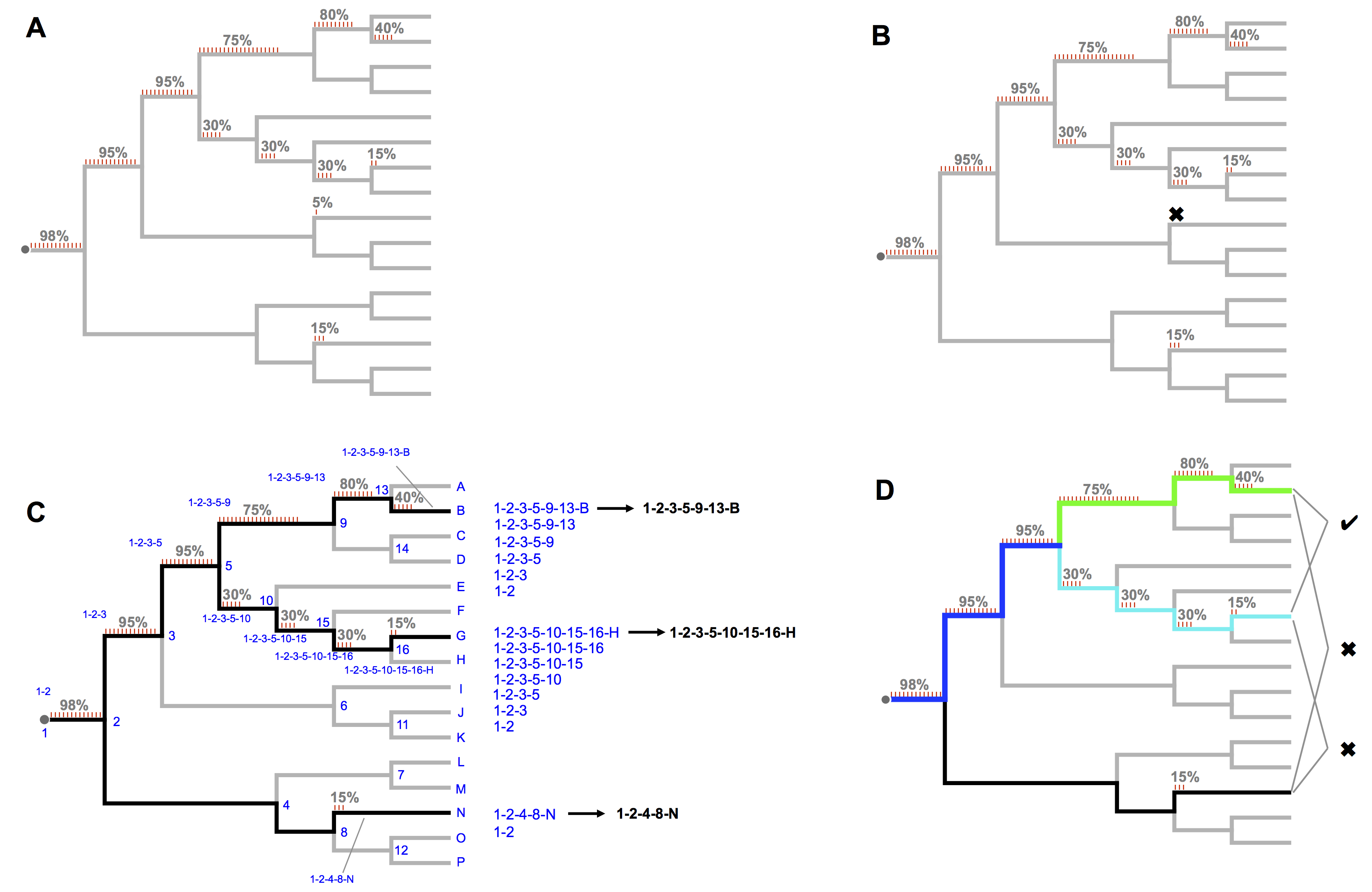

Supplement: S2 Fig — (A) Map the SNVs (red vertical lines) of a sample to the reference phylogeny. (B) Exclude branches with low SNV coverage (<10%). (C) Assemble mapped branches into candidate evolutionary paths (black thick lines). Strings above/below branches indicate evolutionary routes of corresponding branches. (D) Determine authentic pairs of paths (colored lines) from all possible combinations. (TIFF) [file pone.0159029.s002.tiff]

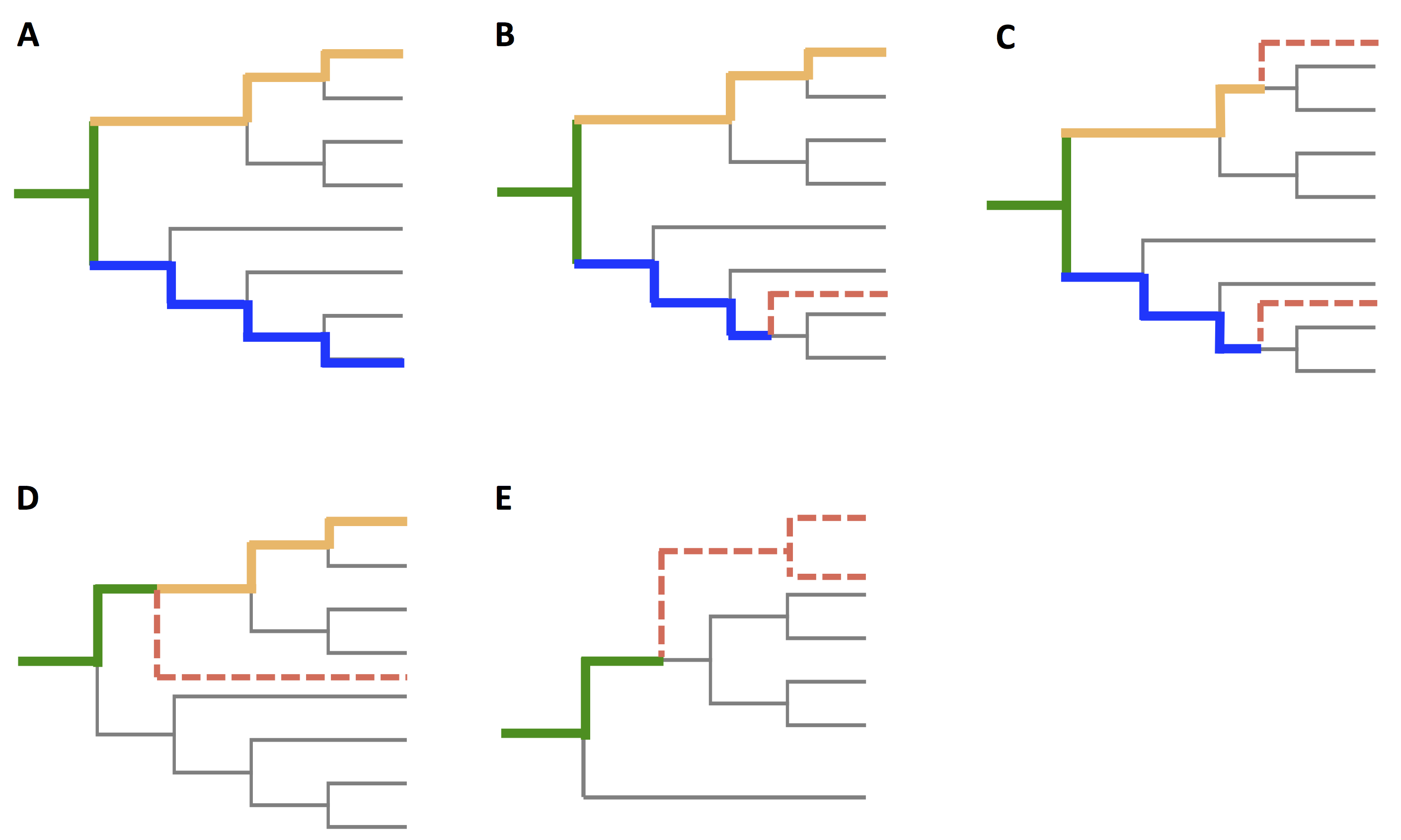

Supplement: S3 Fig — The two unique paths after divergence are completely (A), or partially (B, C) covered by the database. In other cases, one (D) or both (E) unique paths are not covered by the database. Thick colored lines represent path segments covered by the database. Red dash lines represent path segments not included in the database. (TIFF) [file pone.0159029.s003.tiff]

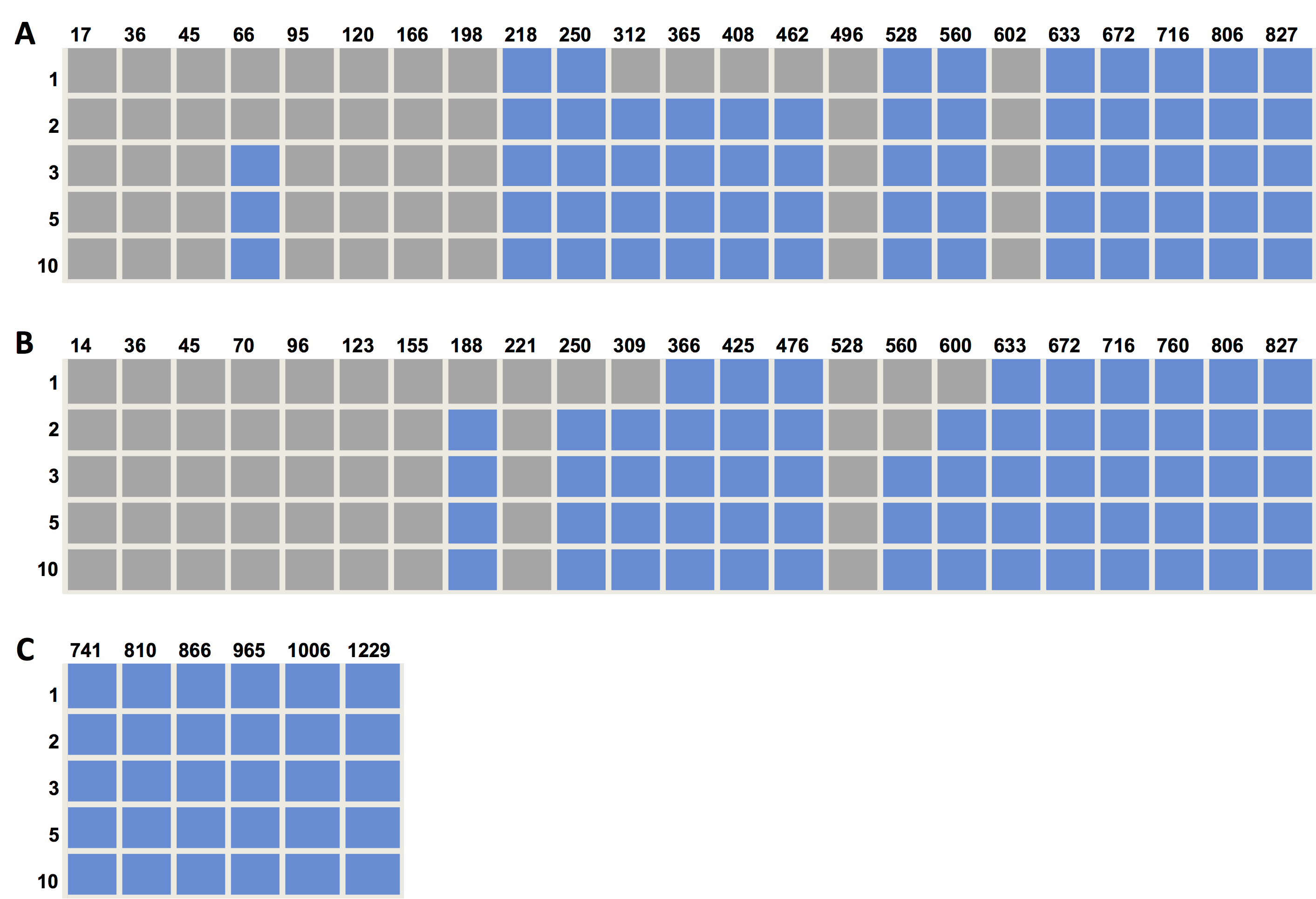

Supplement: S4 Fig — Three panels represent simulated mixed infections by strains within Lineage 2 (A) or Lineage 4 (B), or by strains of different lineages (C). For each panel, the numbers above the first row represent the genomic distance between two strains. The numbers left to the first column represent the depth of the minor strains. The gray and blue cells indicate failure and success of detection respectively. (TIFF) [file pone.0159029.s004.tiff]
